# Observing the Viscous Relaxation Process of Silica Optical Fiber at ~1000 °C Using Regenerated Fiber Bragg Grating

**DOI:** 10.3390/s19102293

**Published:** 2019-05-17

**Authors:** Zhiru Cui, Jianhui Gong, Chen Wang, Nana Che, Yanshuang Zhao, Quan Chai, Haifeng Qi, Elfed Lewis, Jing Ren, Jianzhong Zhang, Jun Yang, Libo Yuan, Gang-Ding Peng

**Affiliations:** 1 Key Lab of In-Fiber Integrated Optics of Ministry of Education, Harbin Engineering University, Harbin 150001, China; cuizhiru@hrbeu.edu.cn (Z.C.); gongjianhui@hrbeu.edu.cn (J.G); wangchen123@hrbeu.edu.cn (C.W.); chenana906@hrbeu.edu.cn (N.C.); zhangjianzhong@hrbeu.edu.cn (Y.Z.); chaiquan@hrbeu.edu.cn (Q.C.); Elfed.Lewis@ul.ie (E.L.); ren.jing@hrbeu.edu.cn (J.R.); yangjun@hrbeu.edu.cn (J.Y); yuanlibo@hrbeu.edu.cn (L.Y.); 2 Laser Research Institute, Qilu University of Technology (Shandong Academy of Science), Jinan 250100, China; qihf@sdlaser.cn; 3 Optical Fiber Sensors Research Center, University of Limerick, V94 T9PX Limerick, Ireland; 4 Photonics and Optical Communications Group, School of Electrical Engineering, University of New South Wales, Sydney, NSW 2052, Australia; g.peng@unsw.edu.au

**Keywords:** regenerated fiber Bragg grating, viscous relaxation, optical fiber, viscosity

## Abstract

A regenerated fiber Bragg grating (RFBG) in silica fiber was used to observe the viscous relaxation process of the host silica fiber at high temperatures of around 1000 °C. Two factors, preannealing time and loaded tension, which affect viscous relaxation, were observed. When an RFBG is stretched after a longer preannealing, the measured viscosity of the optical fiber was observed to reach equilibrium faster, which means that preannealing accelerates viscous relaxation. A similar acceleration phenomenon was also observed when a larger load was applied to stretch the optical fiber, although the acceleration effect of loaded tension was not as strong as in the preannealing case. The results play an active role in establishing effective optical-fiber devices for application in high-temperature environments.

## 1. Introduction

Viscosity is a characteristic parameter that describes the properties of a material [1]. It is a key parameter throughout the entire process of glass production and is often used as an important indicator for controlling and evaluating the process and performance of glass production. Different glass-forming methods and speeds relate to their viscosities. During the annealing process, the viscosity of the glass plays an important role in helping to eliminate internal stress in the glass [2]. The main factors affecting viscosity are chemical composition and temperature, which are also related to heating time within the transition-temperature range [3,4]. The variation of the viscosity of silica glass with time at a constant temperature is described as the relaxation process. The viscosity of silicate glass is studied by applying a pressure scheme [5], and the viscosity of glass fibers is measured using stretching or bending schemes [6,7].

Due to its excellent performance, silica glass fiber is widely used in telecommunications, as well as sensing, including the fields of oil and gas production, aerospace, and nuclear and industrial chemical plants. The applications of fiber sensors in high-temperature environments have become more widespread and therefore more widely reported in the literature. Viscosity and its relaxation of optical fibers at high temperatures have become important in their fabrication, and are therefore of greater significance [8,9,10]. A fiber-bending method can be used to study viscosity and relaxation below the glass transition temperature and to analyze the relationship between the glass structure and the relaxation mechanism. At the glass transition temperature, the viscosity of the drawn and preannealed glass fibers increases with increasing preannealing temperature and time [11,12,13]. Sakaguchi et al. [14] used a cantilever beam-bending method to obtain viscosity values for fibers in the temperature range of 1050 to 1200 °C. This result was several orders of magnitude lower than the viscosity of conventional silica glasses, and suggests that structural relaxation occurred during heat treatment. Shao et al. [15] used regenerated fiber Bragg gratings (RFBGs) to characterize the viscosity of the fiber, and obtained the viscosity values of the fiber at every 25 °C in the range of 1000 to 1150 °C. This article focuses on the viscosity of the fiber at different temperatures and derives the annealing and strain temperatures for an optical fiber, but without considering the viscous relaxation process of the fiber during heat treatment. In order to more thoroughly investigate changes in the viscosity of optical fibers, some work is presented to investigate the relaxation process during the interval covering the prepared stage to the onset of structural equilibrium during heat treatment.

In this paper, focus is placed on the viscous relaxation processes at the temperature of around 1000 °C using infiber RFBGs. The relaxation processes related to the applied tension and the preannealing time on the silica fiber are studied in detail. It provides an effective way for observing the viscous relaxation process of a fiber in a high-temperature environment, which, in turn, is useful for optical-fiber sensor devices designed for high-temperature applications [16,17,18,19,20,21].

## 2. Experimental Procedure

**Figure 1:**
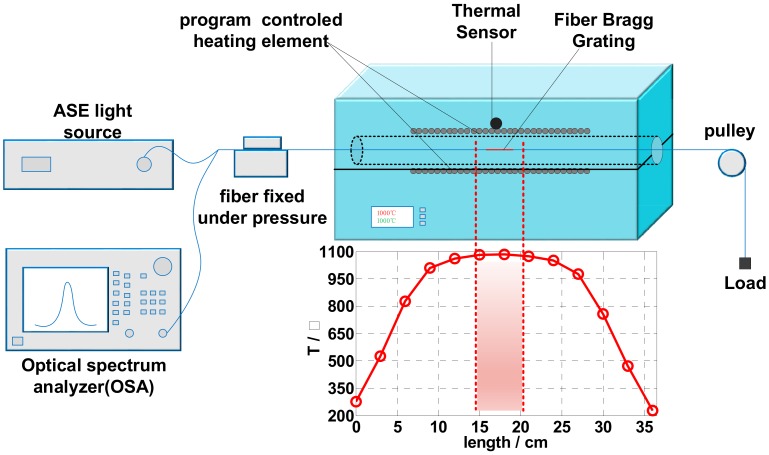
Experimental device and temperature distribution in the tube furnace.

Infiber RFBGs are very stable in high temperatures, and can therefore be used to observe the viscous relaxation processes in the single-mode fiber of SMF-28, based on tensile experiments at 1000 °C, as shown in Figure 1.

The experimental setup used to detect the viscous relaxation processes of the optical fiber is shown in Figure 1, including an amplified spontaneous emission (ASE)-based broadband source (A-0002, Shenzhen Hoyatek Co., Ltd., Shenzhen, China), an optical spectrum analyzer (OSA, AQ6317C, Yokogawa Electric Corporation, Tokyo, Japan), and the tension load system. A tube furnace (T1250S/T1225S, Henan Chengyi Laboratoty Equipment Co., Ltd., Zhengzhou, China) of 37 cm in length was used to provide the high-temperature environment. Its temperature distribution includes a uniform temperature zone in the middle of the tube for an RFBG of ~1 cm in length. The tension-induced strain in the optical fiber was monitored using the Bragg wavelength shift of the RFBG.

**Figure 2:**
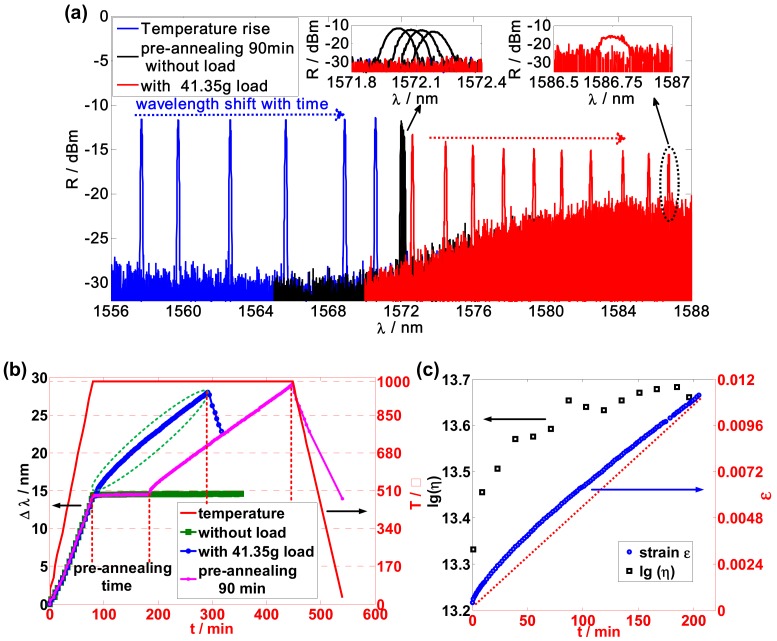
(**a**) Sample spectra of a regenerated fiber Bragg grating (RFBG) during the entire experimental process: Blue lines, temperature-rise process of about 80 min, then maintained constant at 1000 °C; black lines, preannealing for 90 min without load; red lines RFBG spectrum with 41.35 g load; (**b**) Wavelength shift during experimental process. Three representative experiment situations are shown; (**c**) Strain ε and lg(*η*) of regenerated grating with a 41.35 g load.

First, the uncoated-seed FBGs were placed in the uniform temperature zone of the furnace, as shown in Figure 1. Temperature was linearly increased to 1000 °C at a rate of 15 °C/min and thereafter maintained constant. After the seed FBGs sufficiently regenerated and the spectrum of RFBGs were stabilized, the RFBGs were allowed to stretch. In this experiment, the RFBGs were all regenerated by seed FBGs in the same optical fiber at 1000 °C and exposing them to a prior similar thermal process. The RFBGs in the uniform temperature region were stretched, and the entire process of the Bragg wavelength was recorded using the OSA, as shown in Figure 2a. The blue lines in Figure 2a show the temperature-rise process; the black and red lines show the spectrum of RFBG with no load and 41.35 g load at 1000 °C, respectively.

The relationship between the Bragg wavelength shift and time is shown in Figure 2b. The red line represents the temperature change in the middle of the tube. The Bragg wavelength shift of three RFBGs is represented by the green, blue, and pink lines in Figure 2b. During the temperature-rise process, no loading was applied to the RFBGs. Following the temperature-rise process, the three RFBGshad no load applied, load, and a delayed load, and their results are shown as the green, blue, and pink lines, respectively, in Figure 2b. The delayed time is labeled as the preannealing time as shown in Figure 2b. The Bragg wavelength shift can be ignored when the temperature is a constant and no tension is applied on the optical fiber (no load), which is shown as the green line in Figure 2b.

At room temperature, the Bragg wavelength of the FBG shifted to a fixed value when constant tension was applied, and because the optical fiber is an elastic body. This relates to the tension-induced strain as *ε* = Δ*λ_B_*/[*λ_B_*(1 − *p*_e_)] [15]. However, an optical fiber becomes a viscoelastic body at high temperatures. According to the simple Maxwell model [3,12], the relationship between stress, strain, and time is *ε* = *σ*_0_/*E* + *σ*_0_·*t*/*η*. A linear relationship between strain and time exists when fixed stress is applied on a viscoelastic body where *η* is the viscosity and *σ*_0_ is the applied stress, which can be obtained as *σ*_0_ = *m*g/(π*r*^2^), where g = 9.8 m/s2, *r* = 62.5 μm, and *m* is the load mass. *E* is the Young’s modulus of the optical fiber and is constant at 1000 °C. Viscosity can be evaluated using *η* = *σ*_0_/(Δ*ε*/Δ*t*). Δ*ε*/Δ*t*, and is obtained as the slope using linear-regression analysis between ε and t, as shown in Figure 2c.

Viscous relaxation is a process of material-structure alteration and tends toward a balance. In this process, the viscosity of the optical fiber changes with annealing time and eventually settles to a stable value. Figure 2b,c shows the enlarged spectrum and measurement results of an RFBG applied with a load of ~41.35 g. The wavelength shift was not linearly related to time at the beginning but later became much more linear. Strain–time characteristics were divided into different segments, and each segment was individually characterized using a linear fit. Viscosity was calculated and results are shown as the black circles in Figure 2c, where the viscous relaxation process was readily observed.

## 3. Results

**Figure 3:**
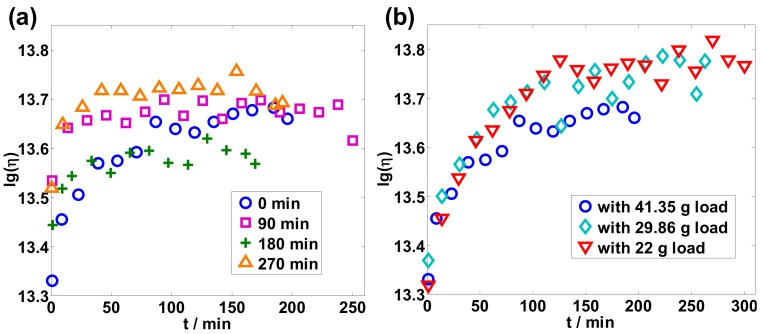
Logarithmic plot of viscosity: (**a**) Viscosity for different preannealing times; (**b**) viscosity with different loads.

Viscosity was measured for different tension loads and different preannealing times, and results are shown in Figure 3a,b, respectively. The viscous relaxation process could be clearly observed, and the log of the viscosity became stable at a value between 13.6 and 13.8, which corresponds to an absolute viscosity value of ~10^13^ Pa·s, which agrees well with previously reported results [13,14,15].

**Figure 4:**
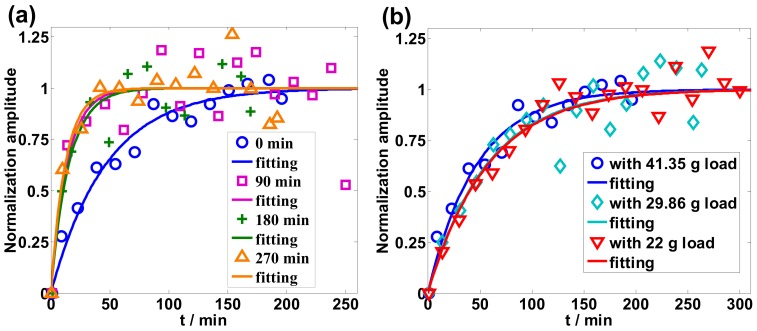
(**a**) Normalization of different preannealing times; (**b**) normalization of different loads.

In order to describe the speed of the viscous relaxation process, the experiment data were normalized as shown in Figure 4, as *n* = (*η* − *η*_min_)/(*η*_0_ − *η*_min_). *η*_0_ is the average stabilized viscosity and *η*_min_ is the minimum viscosity value in Figure 3. The following equation was used to fit the normalized experiment data [13]:

(1) $$n=1-\exp(-\frac{t}{\tau_{0}})$$

where *n* is the normalization amplitude, and *τ*_0_ a fitting parameter that can be used to characterize the speed of the viscous relaxation process, as shown in Table 1 and Table 2. In Figure 4a, the R-square values of the four fitting lines are 0.9615, 0.7464, 0.8169, 0.8977, corresponding to the preannealing times of0, 90, 180, and 270 min, respectively. These values show a good fitting relationship between *n* and *t*. Preannealing therefore assists the viscous relaxation process, as shown in Table 1, which can be established by comparing the viscosity data with and without the preannealing time of 90, 180, and 270 min in Figure 3a and Figure 4a. It is therefore clear that viscous relaxation was sped up by preannealing because the relaxation process was introduced by heat treatment.

Viscosity saturated faster for the measurement with a larger load, which can be seen from the relationship between *τ*_0_ and the applied load on the optical fiber in Table 2, even though the relationship is not as clear as in the case of the preannealing process shown in Table 1. The R-square values of three fitting lines in Figure 4b are 0.9615, 0.8821, 0.9441, and correspond to three different loads. When a larger load is applied, larger tensile stress is exerted on the silica optical fiber, and acceleration of the viscous relaxation process occurs.

## 4. Discussion

The tensile experiment in this paper showed good repeatability. However, despite the use of gratings fabricated in the same batch, it is difficult to ensure that their history during writing and even the fiber-drawing process is exactly the same. Therefore, the viscosity of each regenerated grating could be slightly different. The phenomenon of the viscous relaxation process has been comprehensively studied, but the explanation of the fundamental relaxation mechanism should be studied further. Factors affecting the viscosity of silica glass include chemical composition, temperature, and annealing time. In the presented experiments, the viscous relaxation process could be clearly observed, and viscosity became stable at the level of ~10^13^ Pa·s at a constant high temperature ~1000 °C. It also suggested that both the equilibrium rate and the equilibrium viscosity of silica glass fibers can be influenced by preannealing and tension axial stress. Viscosity had time-dependent change that could be attributed to the progress of structural relaxation during heating [13,22]. Sakaguchi et al. [14] determined that viscosity increases with increasing time during heating, during which time the glass structure of the fiber relaxes to an equilibrium state, meaning that viscosity also reaches equilibrium for the attained temperature values.

Preannealing reduces relaxation time and equilibrates the structure. Previously reported results [13] showed that viscosity increases with increasing preannealing temperature and time. Heat treatment increases the local crystallinity and therefore assists in the reduction of local stresses in the core, leading to more homogeneous fibers [23]. With increasing time, high-temperature preannealing results in a tighter microstructure of the silica optical fiber, thus giving a change of viscosity, as the viscous relaxation process and the longer preannealing time correspond to a smaller *τ*_0_.

The viscous relaxation process of the fiber also exhibited tensile-related viscous relaxation relationship that is probably related to the tensile force affecting the microstructure of the fiber in the heat-treatment environment [24,25,26]. Mechanical stretching was reported that can also induce structural changes and the energy enhancement of the glass fiber [27]. It is also assumed that the as-drawn glass fiber has many internal structural distortions and distributions of bond angles and bond length compared with the preannealed glass fiber [13]. Therefore, it is further assumed that tension-induced axial stress caused by load stretching is effective for causing the viscous relaxation of the silica optical fiber, and hence manifests as a tension-related phenomenon. This phenomenon can possibly be related to the occurrence of stress relaxation and structural relaxation. It was reported that the surface structural relaxation of silica glass was accelerated by the applied tensile stress, and this was experimentally confirmed using IR reflection spectroscopy; structural relaxation is promoted by tensile stress while it is suppressed by compressive stress [28,29]. In addition, the relaxation of the net core stress of the optical fiber is proportional to the drawing force [30]. The applied tensile stress can therefore result in relaxation enhancement and reduce relaxation time [31].

## 5. Conclusions

The viscous relaxation process of the optical fiber at 1000 °C was experimentally assessed using regenerated optical-fiber Bragg gratings. Clear time-dependent change in viscosity was observed and understood as a viscous relaxation process. The log of the viscosity of the optical fiber during the viscous relaxation process was obtained as being in the range of 13.2 to 13.8, stabilizing between 13.6 and 13.8. The equilibrium viscosity in this manuscript was consistent with previously reported values for silica optical fibers. Furthermore, investigations revealed that two factors, preannealing and load-induced tension, accelerate the viscous relaxation process of optical fibers. The significant difference in the experiment results between no preannealing and preannealing for 270 min indicates that acceleration induced by preannealing is clearly a genuine phenomenon. The experiment results also show that application of a larger load induces the acceleration of viscous relaxation, which was established through a comparison of three different loads. In conclusion, the change in viscosity indicates that viscous relaxation occurs during heat treatment, and the structure of the optical fiber was allowed to sufficiently relax to equilibrium provided the experimental conditions are well-controlled.

## Figures and Tables

**Table 1:** Summary of exponential fitting parameter *τ*_0_ with different preannealing times.

Preannealing Time (min)	0	90	180	270
*τ*_0_	47.17	13.90	15.55	12.35

**Table 2:** Summary of exponential fitting parameter *τ*_0_ with different loads.

Load (g)	22.2	29.86	41.35
*τ*_0_	57.97	56.88	47.17
